# Secondary cancers after carbon‐ion radiotherapy and photon beam radiotherapy for uterine cervical cancer: A comparative study

**DOI:** 10.1002/cam4.4622

**Published:** 2022-03-23

**Authors:** Yuki Nitta, Hiroto Murata, Noriyuki Okonogi, Kazutoshi Murata, Masaru Wakatsuki, Kumiko Karasawa, Shingo Kato, Shigeru Yamada, Takashi Nakano, Hiroshi Tsuji

**Affiliations:** ^1^ QST Hospital, National Institutes for Quantum Science and Technology Chiba Japan; ^2^ Department of Radiation Oncology Gunma University Graduate School of Medicine Maebashi Japan; ^3^ Department of Radiation Oncology Saitama Cancer Center Saitama Japan; ^4^ Department of Radiation Oncology Tokyo Women's Medical University School of Medicine Tokyo Japan; ^5^ Department of Radiation Oncology Saitama Medical University International Medical Center Hidaka Japan; ^6^ Quantum Medical Science Directorate National Institutes for Quantum Science and Technology Chiba Japan

**Keywords:** carbon‐ion radiotherapy, chemoradiotherapy, neoplasms, radiation oncology, second primary, uterine cervical neoplasms

## Abstract

**Background:**

There are limited studies on the risk of secondary cancers after carbon‐ion radiotherapy (CIRT). We assessed the incidence of secondary cancers in patients treated with CIRT for cervical cancer. We also evaluated the incidence of secondary cancers in patients who received standard photon radiotherapy (RT) throughout the same period.

**Methods:**

This retrospective study included patients with cervical cancer who underwent curative RT at our hospital. All cancers discovered for the first time after RT were classified as secondary cancers. To compare the risk of secondary cancers among cervical cancer survivors to the general population, standardized incidence ratios (SIRs) were calculated.

**Results:**

The analysis included a total of 197 and 417 patients in the CIRT and photon RT groups, respectively. The total person‐years during the observation period were 1052.4 in the CIRT group and 2481.5 in the photon RT group. The SIR for all secondary cancers was 1.1 (95% confidence interval [CI], 0.6–2.1) in the CIRT group and 1.4 (95% CI, 1.0–2.1) in the photon RT group. The 10‐year cumulative incidence of all secondary cancers was 9.5% (95% CI, 4.0–21.5) in the CIRT group and 9.4% (95% CI, 6.2–14.1) in the photon RT group. The CIRT and photon RT groups were not significantly different in incidence (*p* = 0.268).

**Conclusions:**

The incidence of secondary cancers after CIRT for cervical cancer was similar to that after photon RT. Validation of our findings after long‐term observation is warranted.

## Graphical abstract

We investigated the incidence of secondary cancers in patients treated with carbon‐ion (C‐ion) radiation therapy (RT) or photon RT for cervical cancer. The incidence of secondary cancers after C‐ion RT for cervical cancer was similar to that after photon RT.

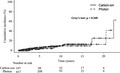

## 1 INTRODUCTION

Cervical cancer, mainly caused by chronic infection with high‐risk human papillomavirus, is common cancer in women.^1^ The number of new cervical cancer cases are estimated to be approximately 569,000 around the world annually.^2^ According to the guidelines of the national comprehensive cancer network, radiation therapy (RT) /concurrent chemo‐RT (CCRT) is the standard care for cervical cancer, except for stage IA cancers.^3^ With recent technological advancements, RT, including three‐dimensional image‐guided brachytherapy (3D‐IGBT), has become a necessary component of cervical cancer treatment.^4^, ^5^ Recent studies involving 3D‐IGBT have shown favorable clinical outcomes for cervical cancer.^6^, ^7^, ^8^ According to a recent multi‐institutional prospective study, the overall 5‐year actuarial local control rate was 92%, with limited severe toxicity to healthy organs.^6^

Carbon‐ion radiotherapy (CIRT) has two distinct advantages over conventional RT: dose localization and biological efficacy due to high linear energy transfer.^9^, ^10^ CIRT has been used to treat various types of cancers, including uterine cervical cancer.^11^ Even with CCRT, including 3D‐IGBT, the clinical results for adenocarcinoma of the uterine cervix remain poor.^12^, ^13^, ^14^ In comparison, CIRT showed favorable clinical outcomes for uterine cervix adenocarcinoma, and those results were validated in a recent multi‐institutional study.^15^, ^16^, ^17^

As treatment outcomes for cervical cancer improve, the need to control late adverse events is an important clinical issue. Typical late adverse events after administering RT/CIRT for cervical cancer include rectal complications, bladder complications, and insufficiency bone fractures.^6^, ^17^, ^18^, ^19^, ^20^ Secondary cancers are also an adverse event that deserves posttreatment attention. A previous study reported a significant 1.2‐fold (95 percent confidence interval [CI], 1.1–1.4) increased risk of developing secondary cancers in patients with cervical cancer treated with conventional definitive RT compared to the general population, equating to a 1.6‐percent excess risk per person per decade of follow‐up.^21^ Mohamad et al. recently found that CIRT for prostate cancer was associated with a lower risk of secondary cancers when compared to photon RT.^22^ Considering its dose‐localization properties, CIRT may reduce the risk of secondary cancers. However, the potential risk of secondary cancers may nonetheless increase owing to the effects of high LET radiation on normal tissues and the generation of neutron radiation during CIRT.

Hence, we assessed the incidence of secondary cancers in patients treated with CIRT for cervical cancer at our institution. Moreover, we also compared the incidence with secondary cancers in patients who received conventional photon RT during the same period.

## 2 MATERIALS AND METHODS

### 2.1 Participants and study design

Patients with uterine cervical cancer treated at the National Institutes for Quantum Science and Technology (QST) hospital who received CIRT or photon RT between January 1, 1995, and March 31, 2016, were included in this retrospective study. The patients in the present study all had a histological diagnosis of cervical cancer and underwent definitive RT. At our hospital, all patients with cervical cancer undergo gynecological examinations, pelvic magnetic resonance imaging, and chest to abdominal computed tomography (CT) before the treatment, regardless of the treatment strategy. Thus, in the present study, there was no between‐group difference in pretreatment screening. Patients with synchronous malignancies or distant metastases, those who received RT for the para‐aortic lymph node region, those who received RT as postoperative irradiation, and those unable to complete the scheduled RT were excluded. The institutional ethics review board of the QST approved this retrospective study (number 18–012). Because of the retrospective nature of the present study, the requirement for written informed consent was waived. Instead, a document declaring an opt out policy allowing any of the patients and their families to refuse to participate in the study was posted on our institution's website.

### 2.2 Treatment regimens

The detailed regimens of CIRT and photon RT at our hospital have been described elsewhere.^15^, ^16^, ^23^ Both CIRT and photon RT were administered with curative intent, including whole pelvic irradiation and local tumor irradiation. However, CIRT was performed as a clinical trial and was not combined with intracavitary brachytherapy. Patients were younger than 70 years, and those who could tolerate this regimen received 40 mg/m^2^ of cisplatin weekly as concurrent chemotherapy. The median CIRT dose was 72.0 Gy (relative biological effectiveness [RBE] range, 52.8–74.4) delivered over a median of 20 fractions over 5 weeks (range, 20–24 fractions over 5–6 weeks). Whole pelvic irradiation and central shielding were used in photon RT, with a dose of 2.0 Gy or 1.8 Gy per fraction and a median dose of 50.0 Gy (range, 45.0–50.6). Patients with gross lymph node metastases received a 6.0–10.0 Gy boost RT at the site of the metastasis. Brachytherapy was performed using Ir‐192 with a point A prescription of 6.0 Gy in each fraction, for a total of four fractions (microSelectron HDR; Elekta Instrument AB, Stockholm, Sweden). In‐room CT was introduced in 2001, and we shifted from two‐dimensional planning to 3D‐IGBT.

### 2.3 Data collection

We collected information from the QST database and medical records on patient age, medical history, smoking and alcohol habits, characteristics and treatment of cervical cancer, and posttreatment course. The Charlson Comorbidity Index was applied to classify patient comorbidities.^24^ For the first 2 years, follow‐up was done every 1–3 months, then every 3–6 months for the next 3 years; after 5 years, follow‐up was done every 6–12 months, in principle. Data collection methods were similar to that employed in our previous study.^22^ In brief, medical, radiology, surgical, and pathology reports were combed for information on secondary cancers. Patients who did not receive face‐to‐face follow‐up were mailed a yearly questionnaire with specific questions about cervical cancer recurrence, adverse events after treatment, and the development of secondary cancers. Additional information on secondary cancers was gathered by calling other doctors, hospitals, and patients or their families. With the Ministry of Justice's approval, missing patient data were supplemented from the Japanese nationwide registry that includes the date and cause of death.

Secondary cancers were defined as all cancers observed for the first time after the initiation of RT. Accordingly, all uterine cancers that developed after RT initiation, except those that were recurrences (as confirmed histologically), were registered as secondary cancers of the uterus. The observation period was defined as the time between the first RT session for cervical cancer and death, the second cancer diagnosis, or the last observation date before May 31, 2018, whichever came first.

To compare the risk of secondary cancers among cervical cancer survivors to the general population, standardized incidence ratios (SIRs) were calculated. SIRs were computed by dividing the number of secondary cancer cases observed by the expected number of cases in each cancer location. Using data from the National Cancer Center in Japan, the expected number of secondary cancer patients were determined by applying site‐specific cancer incidence rates from the general female population to the corresponding person‐years of patients with cervical cancer in the cohort.^25^

### 2.4 Statistical analysis

The Mann–Whitney *U* test or Welch's *t*‐test was employed for continuous variables, while the chi‐square test with Yates' adjustment was used for nominal variables. The cumulative incidence of secondary cancers in the CIRT and photon RT groups were compared using Gray's test. Univariate and multivariate analyses of each factor's cumulative incidence of secondary cancers were conducted using the Fine–Gray proportional hazards regression model. The following factors were used in the univariate analysis: types of RT (CIRT or photon), age, smoking and alcohol habits, Charlson Comorbidity Index, tumor histology, stage of the disease, concurrent chemotherapy, and year of treatment. Only factors with a *p*‐value <0.1 were used for multivariate analysis. We used propensity score matching to reduce the impact of treatment selection bias. With a caliper width of 0.2, propensity score matching was an optimal one‐to‐one match. All statistical tests were two‐sided, and all comparisons were considered statistically significant if the *p*‐value was <0.05. EZR version 1.54, a statistical software based on R and R Commander, was used for all analyses.^26^

## 3 RESULTS

**FIGURE 1:**
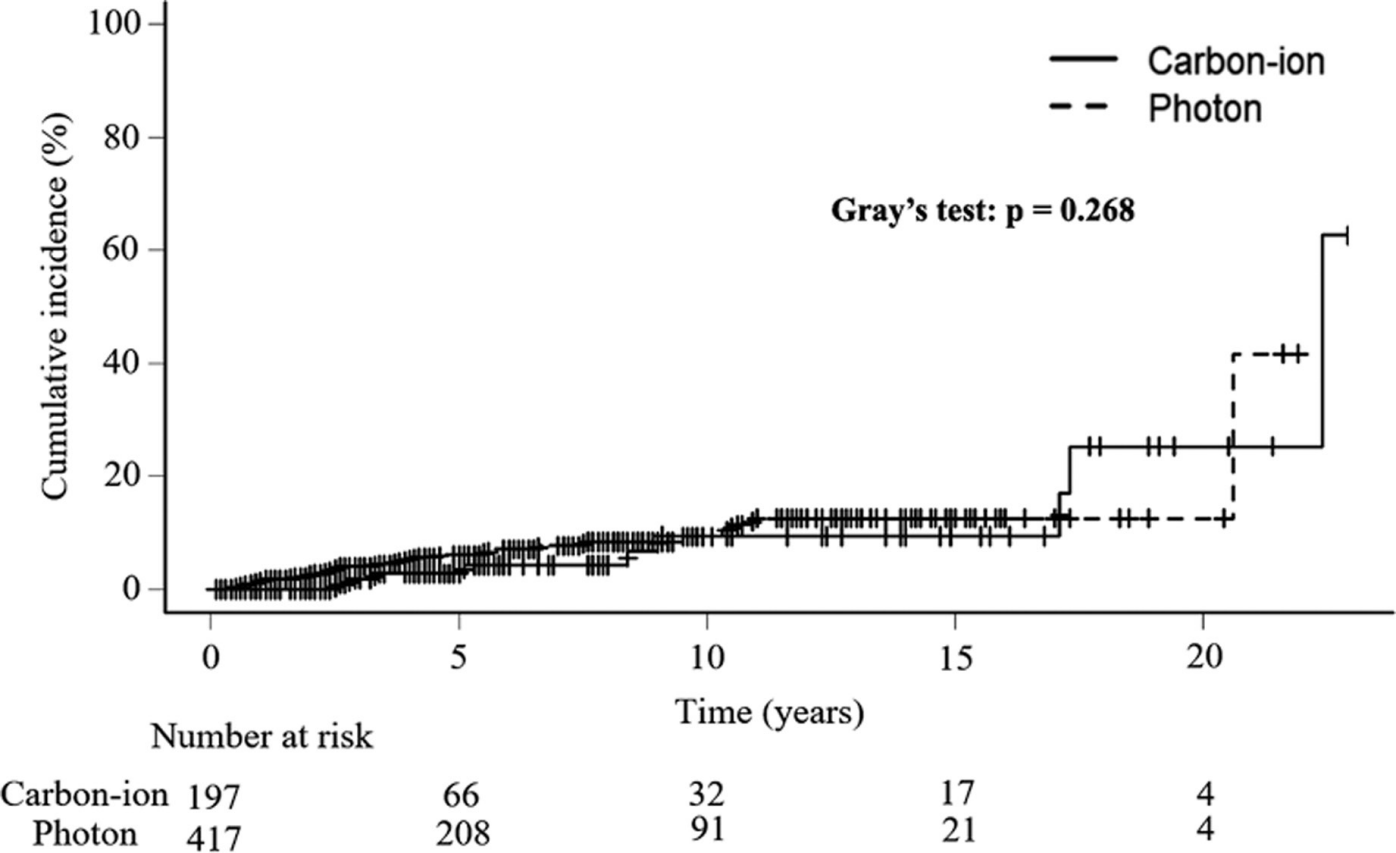
Cumulative incidence of overall secondary cancers after radiotherapy. The solid line indicates the incidence after carbon‐ion radiotherapy. The dotted line indicates the incidence after photon radiotherapy

The present study included 197 patients in the CIRT group and 417 patients in the photon RT group. The cohort's baseline characteristics are listed in Table 1. The median observation period was 3.2 years in the CIRT group and 4.9 years in the photon RT group. The observed person‐years were 1052.4 in the CIRT group and 2481.5 in the photon RT group. The crude incidence of secondary cancers was 9 (4.6%) in the CIRT group and 28 (6.7%) in the photon RT group. The incidence per 1000 person‐years was 8.6 in the CIRT group and 11.3 in the photon RT group. The minimum time to develop any secondary cancer was 0.4 years, and the maximum time was 22.4 years. The median time to develop secondary cancers was 8.4 years in the CIRT group and 3.0 years in the photon RT group. Table S1 lists all patients with secondary cancer in the CIRT and photon RT groups. Figure 1 shows the cumulative incidence curves for all secondary cancers in the CIRT and photon RT groups. The incidence of secondary cancers did not differ significantly between the CIRT and photon RT groups (*p* = 0.268). The 10‐year cumulative incidence of all secondary cancers was 9.5% (95% confidence interval [CI], 4.0–21.5) in the CIRT group and 9.4% (95% CI, 6.2–14.1) in the photon RT group.

**TABLE 1:** Characteristics of patients in the carbon‐ion radiotherapy and photon radiotherapy cohort

	Carbon‐ion (*n* = 197)	Photon (*n* = 417)	*p‐*value
Age (years)			
Median (IQR)	56 (47–66)	66 (54–75)	<0.001
Range	26–85	30–89	
≤40	22 (11%)	26 (6%)	
41–50	53 (27%)	51 (12%)	
51–60	42 (21%)	81 (19%)	
61–70	47 (24%)	102 (24%)	
71–80	32 (16%)	116 (28%)	
>80	1 (1%)	41 (10%)	
Smoking history			
Never	143 (73%)	304 (73%)	0.059
Ever	54 (27%)	102 (24%)	
Unknown	0 (0%)	11 (3%)	
Alcohol habit			
No or Unknown	134 (68%)	317 (76%)	0.046
Yes	63 (32%)	100 (24%)	
Charlson Comorbidity Index			
0–1	181 (92%)	386 (93%)	0.891
2+	16 (8%)	31 (7%)	
Cervical cancer histology			
Squamous cell carcinoma	86 (44%)	378 (91%)	<0.001
Adenocarcinoma	95 (48%)	34 (8%)	
Adenosquamous carcinoma	16 (8%)	5 (1%)	
FIGO stage (2008)			
I–II III–IVA	61 (31%) 136 (69%)	235 (56%) 182 (44%)	<0.001
Concurrent chemotherapy			
No	139 (71%)	305 (73%)	0.568
Yes	58 (29%)	112 (27%)	
Calendar year of treatment			
1995–2005	96 (49%)	283 (68%)	<0.001
2006–2016	101 (51%)	134 (32%)	
Follow‐up (years)			
Median (IQR) for all patients	3.2 (1.9–5.9)	4.9 (2.0–9.1)	0.024
Median (IQR) for surviving patients	5.2 (4.2–11.3)	7.2 (4.1–11.9)	0.457

Abbreviations: FIGO, The International Federation of Gynecology and Obstetrics; IQR, Interquartile range.

Table 2 compares the SIRs of specific secondary cancers in the CIRT and photon RT groups to that in the general population. The SIR of all secondary cancers was 1.1 (95% CI, 0.6–2.1) in the CIRT group and 1.4 (95% CI, 1.0–2.1) in the photon RT group. There was no statistically significant difference in the incidence of secondary cancers between CIRT and photon RT in the pelvis (*p* = 0.388) or outside the pelvis (*p* = 0.353) (Figure S1). Secondary cancers occurred in the pelvis in seven of nine cases in the CIRT group and eight of 28 cases in the photon RT group. The proportion of secondary cancers occurring inside of the pelvis and the outside of the pelvis differed between the CIRT and photon beam RT groups; the incidence of secondary cancers in the pelvis was significantly higher in the CIRT group than in the photon RT group (*p* = 0.026, chi‐square test).

**TABLE 2:** Risk of second cancers by sites after primary radiotherapy

	Carbon‐ion (*n* = 197)			Photon (*n* = 417)		
	Observed number of patients	Expected number of patients	SIR (95% CI)	Observed number of patients	Expected number of patients	SIR (95% CI)
All subsequent cancers	9	8.2	1.1 (0.6–2.1)	28	19.4	1.4 (1.0–2.1)
Hematological malignancies						
Plasmacytoma	1	0.1	17.9 (2.1–151.8)	0	0.1	0
Solid tumors						
Uterine	2	0.3	7.6 (1.8–32.1)	0	0.6	0
Ovary	1	0.2	4.6 (0.6–34.5)	1	0.5	2.0 (0.3–14.6)
Colon	1	0.8	1.3 (0.2–9.1)	3	1.9	1.6 (0.5–5.1)
Rectum	0	0.2	0	1	0.6	1.8 (0.2–13.3)
Stomach	0	0.7	0	6	1.6	3.8 (1.6–8.7)
Liver	0	0.2	0	2	0.5	3.7 (0.9–15.6)
Pancreas	0	0.3	0	1	0.8	1.3 (0.2–9.7)
Bile duct	0	0.1	0	1	0.2	4.4 (0.6–34.5)
Bladder	1	0.1	10.8 (1.4–85.4)	2	0.2	9.2 (2.0–42.5)
Lung	1	0.7	1.5 (0.2–10.7)	6	1.6	3.8 (1.6–8.7)
Breast	1	1.5	0.7 (0.1–4.7)	1	3.6	0.3 (0.0–2.0)
Hypopharynx	0	0.0	0	1	0.0	100.7 (2.6–3940.8)
Thyroid	0	0.2	0	1	0.5	1.9 (0.3–13.9)
Skin (melanoma)	0	0.0	0	1	0.0	26.7 (2.1–337.2)
Soft tissue (pelvis)	1	0.0	41.3 (4.0–431.9)	1	0.1	17.5 (1.7–183.2)
Inside of the pelvis	7	N/A		8	N/A	
Outside of the pelvis	2	N/A		20	N/A	

Abbreviations: CI, confidence interval; N/A, not available; RT, radiotherapy; SIR, standardized incidence ratio.

Table 3 shows the results of univariate and multivariate analyses of secondary cancer risk factors. The patient's age and calendar year of treatment were associated with the risk of secondary cancers. Regarding the patient's age, the hazard ratio was 1.07 (95% CI, 1.03–1.10) for each additional year of age at the start of treatment. For the calendar year of treatment, the risk of secondary cancers was higher in the recently treated group, with a hazard ratio of 2.72 (95% CI, 1.35–5.48). Other factors, including CIRT or photon RT, smoking, and alcohol habit, did not show any significant differences.

**TABLE 3:** Fine and Gray competing risk regression model (univariate and multivariate analyses) for overall second cancers

Factor	Univariate analysis		Multivariate analysis	
	Hazard ratio (95% CI)	*p*‐value	Hazard ratio (95% CI)	*p*‐value
Carbon‐ion vs. Photon	0.64 (0.30–1.39)	0.26		
Age (continuous)	1.07 (1.03–1.10)	<0.001		
Age (>62 vs. ≤62 years)	2.93 (1.46–5.89)	0.003	3.17 (1.56–6.41)	0.001
Smoking history (ever vs. never & unknown)	1.31 (0.63–2.72)	0.47		
Alcohol habit (yes vs. no & unknown)	0.85 (0.37–1.95)	0.69		
Charlson Comorbidity Index (0,1 vs. 2)	0.85 (0.22–3.37)	0.82		
Cervical cancer histology (AC & ASC vs. SCC)	0.50 (0.35–1.98)	0.68		
FIGO stage (2008) (III–IV vs. I–II)	1.08 (0.54–2.15)	0.83		
Concurrent chemotherapy (yes vs. no)	0.50 (0.20–1.26)	0.14		
Calendar year of treatment (2006–2016 vs. 1995–2005)	2.45 (1.23–4.89)	0.011	2.72 (1.35–5.48)	0.005

Abbreviations: AC, adenocarcinoma; ACC, squamous cell carcinoma; ASC, adenosquamous cell carcinoma; CI, confidence interval; FIGO, The International Federation of Gynecology and Obstetrics.

**FIGURE 2:**
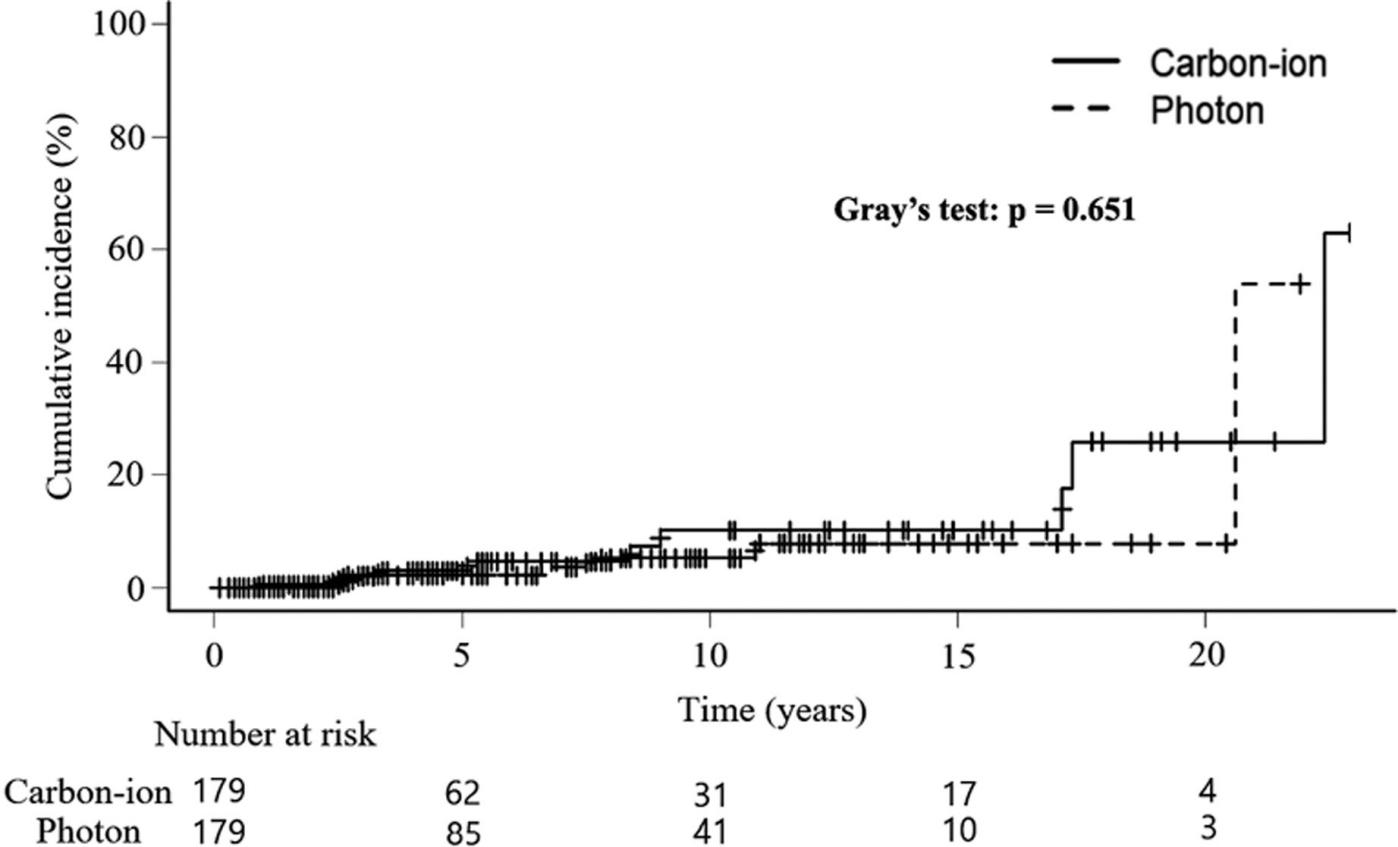
Cumulative incidence with propensity score matching of overall secondary cancers after radiotherapy. The solid line indicates the incidence after carbon‐ion radiotherapy. The dotted line indicates the incidence after photon radiotherapy

We then examined the incidence of secondary cancers in the CIRT and photon RT groups after matching for age and calendar year of treatment conditions. The baseline characteristics of the CIRT and photon RT groups after propensity score matching are shown in Table 4. This matching showed no statistically significant difference in smoking history, alcohol habit, or Charlson Comorbidity Index between the CIRT and photon RT groups. Although the cervical cancer histology and stage were statistically significant between the two groups, the univariate and multivariate analyses showed that these were not risk factors for secondary cancers (Table 3). Thus, propensity score matching was valid in comparing the frequency of secondary cancers in the CIRT and photon RT groups. Figure 2 shows the cumulative incidence curves of secondary cancers after matching. Secondary cancers incidence did not differ significantly between the CIRT and photon RT groups (*p* = 0.651).

**TABLE 4:** Characteristics of patients in the carbon‐ion radiotherapy and photon radiotherapy with the matching of the age and calendar year of treatment

	Carbon‐ion (*n* = 179)	Photon (*n* = 179)	*p* value
Age (years)			
Median (IQR)	57 (49–67)	58 (49–67)	0.930
Range	31–85	31–85	
Smoking history			
Never	131 (73%)	120 (67%)	0.052
Ever	48 (27%)	54 (30%)	
Unknown	0 (0%)	5 (3%)	
Alcohol habit			
No or Unknown	126 (70%)	133 (74%)	0.478
Yes	53 (30%)	46 (26%)	
Charlson Comorbidity Index			
0–1	165 (92%)	172 (96%)	0.177
2+	14 (8%)	7 (4%)	
Cervical cancer histology			
Squamous cell carcinoma	80 (45%)	154 (86%)	<0.001
Adenocarcinoma	85 (47%)	21 (12%)	
Adenosquamous carcinoma	14 (8%)	4 (2%)	
FIGO stage (2008)			
I–II	53 (30%)	102 (57%)	<0.001
III–IVA	126 (70%)	77 (43%)	
Concurrent chemotherapy			
No	130 (73%)	113 (63%)	0.070
Yes	49 (27%)	66 (37%)	
Calendar year of treatment			
1995–2005	96 (54%)	96 (54%)	1.000
2006–2016	83 (46%)	83 (46%)	
Follow‐up (years)			
Median (IQR) for all patients	3.3 (1.9–6.2)	4.4 (2.0–9.7)	0.089

Abbreviations: FIGO, The International Federation of Gynecology and Obstetrics; IQR, Interquartile range.

## 4 DISCUSSION

To the best of our knowledge, the present study is the first to investigate the frequency of secondary cancers in patients treated with CIRT for cervical cancer. It is also the first report to directly compare the risk of secondary cancers after CIRT and photon RT for cervical cancer. RT reportedly increases the incidence of secondary cancers by 1.08–1.43 in the general population.^27^ Regarding secondary cancer incidence after RT for cervical cancer, Arai et al. reported that a significant excess in secondary cancer incidence was observed in organs, such as the rectum and bladder, within the irradiated field.^28^ Thereafter, the risk of developing secondary cancer after photon RT for cervical cancer was reported to be approximately 1.2 times higher than that in the general population.^21^ In the present study, the risk of developing secondary cancers after CIRT for cervical cancer was approximately 1.1 (95% CI, 0.6–2.1) times that of the general population, and the risk of developing secondary cancer after photon RT was approximately 1.4 (95% CI, 1.0–2.1) times that of the general population, consistent with previous reports. The lower limit of the 95% CI for the SIR of secondary cancers in the CIRT group was below 1.0, indicating no apparent difference from the general population. This result may be due to an insufficient number of secondary cancer cases and observation period. In recent years, the usefulness of CIRT for hard‐to‐treat gynecologic tumors, such as malignant melanoma and adenocarcinoma of the uterine cervix, has been demonstrated in systematic reviews.^29^, ^30^ Although the benefits of CIRT seem to outweigh the risk of developing secondary cancer, further follow‐up is warranted to establish the clinical benefits of CIRT for gynecologic tumors.

In the present study, patient age at the start of treatment and the calendar year of treatment were risk factors for the development of secondary cancers. No significant differences were found according to the type of RT (CIRT versus photon RT) or smoking and alcohol habits. In general, cancer incidence increases with age.^25^ In the present study, secondary cancers were defined as all cancers observed for the first time after the start of RT. Therefore, it is reasonable that age at the start of treatment may be a risk factor for developing secondary cancers. However, it is not clear why the calendar year of treatment may be a risk factor for the development of secondary cancers. In the present study, the time for development of secondary cancer was significantly shorter in the group treated in recent years (Table S2). The most reasonable explanation would be that the group treated in recent years has a shorter follow‐up time compared to the group treated earlier. Another possibility is that the technological advancement of medical devices for diagnosis has led to the early detection of secondary cancers. Unfortunately, the present analysis did not cover the details of the medical devices used to screen secondary cancer, but further research with longer follow‐up is necessary.

In an epidemiological study that investigated secondary cancers after treatment for cervical cancer using any type of therapy, Arnold et al. reported that smoking and RT were risk factors for secondary cancers.^31^ This discrepancy may be explained by smoking status classification. A different result may be obtained when using the Brinkman index or changing the classification to former/current smokers. Smoking has adverse effects and may cause various diseases other than secondary cancers,^32^, ^33^ and it is essential to educate patients to quit smoking after treatment. Regarding the chemotherapy effect on secondary cancers, the presence of concomitant chemotherapy was not a significant risk factor for secondary cancers in the present study. The alkylating agent procarbazine has been reported to be a risk factor for secondary cancers of the digestive system in survivors of Hodgkin's lymphoma.^34^ Cisplatin is administered in many types of cancer, and its use is associated with an increased incidence of secondary leukemia,^35^ but to date, little information relating to secondary solid tumors is available. Thus, the risk of secondary cancer with cisplatin may not be as high as that with alkylating agents.

Although there was no statistically significant difference in the incidence of secondary cancers between CIRT and photon RT in and outside the pelvis, it is worth noting that CIRT resulted in fewer secondary cancers outside the pelvis, whereas photon RT resulted in more secondary cancers outside the pelvis. A possible reason is that patients who received CIRT did not receive intracavitary brachytherapy. Lee et al. reported that organ exposure from brachytherapy, including scatter radiation, could increase the risk of secondary cancer. They reported in phantom experiments that brachytherapy could increase the risk of secondary cancers of the stomach, lungs, and breasts away from the pelvis.^36^ Furthermore, Yonai et al. reported that the amount of neutron radiation produced by carbon‐ion beams in the irradiation field is lower than that produced by 6‐MV intensity‐modulated RT or gamma‐ray irradiation of Ir‐192 when standardized by treatment dose and is almost undetectable outside of the irradiation field.^37^ Although it is necessary to consider the possibility that various factors contribute to secondary cancer development, the fact that intracavitary brachytherapy was not applied in CIRT, the distribution of neutrons might have manifested as a low incidence of secondary cancers outside the pelvis in CIRT in the present study.

Mohamad et al. reported a lower risk of secondary cancers with CIRT than with photon therapy in the treatment of prostate cancer.^22^ However, in our study, the risk of developing secondary cancers from CIRT was similar to, yet not lower than that from photon RT. This discrepancy may be due to the difference in the size of the irradiation fields for prostate and cervical cancers. Maraldo et al. reported that the size of the irradiation field was a risk factor for secondary cancer development following photon RT.^38^ For CIRT in cervical cancer, the irradiation field includes the pelvic lymph node volume area; the irradiation size is similar to that of photon RT and much larger than the irradiation field for prostate cancers. Currently, scanning irradiation is achievable with CIRT instead of conventional passive irradiation.^39^ Compared with the conventional passive irradiation of CIRT, scanning irradiation not only contributes to the further improvement of the dose distribution in the irradiation field, but also reduces the neutron flux in the field by 60%–70%, because it avoids the generation of neutrons due to collisions between beamline devices and carbon ions.^40^ Thus, CIRT with scanning irradiation may reduce the risk of secondary cancers in the future.

Our study had several limitations. First, the median follow‐up after treatment was as short as 4.4 years. The number of patients with secondary cancers may increase with longer observation after both photon and CIRT. In fact, the lower limit of the 95% CI for the SIR of secondary cancers in the CIRT group was below 1.0. Therefore, re‐evaluation of the findings after long‐term observation is warranted. Second, while the present study included all cancer cases that occurred after treatment, the clinical objective is to determine the risk of radiation‐induced cancers more specifically. Recently, it has been reported that certain genetic mutations are found in radiation‐induced secondary cancers.^41^ Best et al. reported that two variants at chromosome 6q21 were associated with secondary cancers in survivors of Hodgkin's lymphoma treated with radiation therapy as children but not as adults.^41^ Given the complexity of carcinogenesis, it may still be difficult to identify radiation‐induced secondary cancers among secondary cancers. However, it would be desirable to evaluate the risk of radiation‐induced cancers more specifically by genetic mutations in the future.

In conclusion, we found that the incidence of secondary cancers after CIRT for cervical cancer was comparable to that after photon RT. Validation of our findings after long‐term observation is warranted.

## CONFLICT OF INTEREST

None.

## AUTHOR CONTRIBUTIONS

Conceptualization, YN, HM, and NO; acquisition of data, YN, NO, HM, KM, MW, KK, SK, and TN; analysis and interpretation of data, HM and NO; original draft preparation, YN, and HM; review and editing, NO, SY, TN, and HT. All authors agreed on the order in which their names are listed in the manuscript. All authors have read and agreed to the final version of the manuscript.

## ETHICAL APPROVAL STATEMENT

This study was approved by the institutional ethics review board (number 18–012).

## Supporting information


Table S1‐S2

Figure S1

## Data Availability

Research data are stored in an institutional repository and will be shared, upon request to the corresponding author.

## References

[1] Bosch FX , Lorincz A , Muñoz N , Meijer CJ , Shah KV . The causal relation between human papillomavirus and cervical cancer. J Clin Pathol. 2002;55(4):244‐265. doi:10.1136/jcp.55.4.244 11919208 PMC1769629

[2] International Agency for Research on Cancer, World Health Organization . Cervical cancer: estimated incidence, mortality and prevalence worldwide in 2020. Global Cancer Observatory. Updated January 2021. http://gco.iarc.fr/today/data/factsheets/cancers/23‐Cervix‐uteri‐fact‐sheet.pdf. Accessed October 30, 2021.

[3] National Comprehensive Cancer Network . Clinical practice guideline in oncology, cervical cancer. 2021. Updated [October 26, 2021]. https://www.nccn.org/guidelines/guidelines‐detail?category=1&id=1426. Accessed October 30, 2021.

[4] Haie‐Meder C , Pötter R , Van Limbergen E , et al. Recommendations from Gynaecological (GYN) GEC‐ESTRO Working Group (I): concepts and terms in 3D image based 3D treatment planning in cervix cancer brachytherapy with emphasis on MRI assessment of GTV and CTV. Radiother Oncol. 2005;74(3):235‐245. doi:10.1016/j.radonc.2004.12.015 15763303

[5] Pötter R , Haie‐Meder C , Van Limbergen E , et al. Recommendations from gynaecological (GYN) GEC ESTRO working group (II): concepts and terms in 3D image‐based treatment planning in cervix cancer brachytherapy‐3D dose volume parameters and aspects of 3D image‐based anatomy, radiation physics, radiobiology. Radiother Oncol. 2006;78(1):67‐77. doi:10.1016/j.radonc.2005.11.014 16403584

[6] Pötter R , Tanderup K , Schmid MP , et al. MRI‐guided adaptive brachytherapy in locally advanced cervical cancer (EMBRACE‐I): a multicentre prospective cohort study. Lancet Oncol. 2021;22(4):538‐547. doi:10.1016/S1470-2045(20)30753-1 33794207

[7] Sturdza A , Pötter R , Fokdal LU , et al. Image guided brachytherapy in locally advanced cervical cancer: Improved pelvic control and survival in RetroEMBRACE, a multicenter cohort study. Radiother Oncol. 2016;120(3):428‐443. doi:10.1016/j.radonc.2016.03.011 27134181

[8] Ohno T , Noda SE , Okonogi N , et al. In‐room computed tomography‐based brachytherapy for uterine cervical cancer: results of a 5‐year retrospective study. J Radiat Res. 2017;58(4):543‐551. doi:10.1093/jrr/rrw121 27986859 PMC5766167

[9] Kanai T , Furusawa Y , Fukutsu K , Itsukaichi H , Eguchi‐Kasai K , Ohara H . Irradiation of mixed beam and design of spread‐out bragg peak for heavy‐ion radiotherapy. Radiat Res. 1997;147(1):78‐85. 8989373

[10] Kanai T , Endo M , Minohara S , et al. Biophysical characteristics of HIMAC clinical irradiation system for heavy‐ion radiation therapy. Int J Radiat Oncol Biol Phys. 1999;44(1):201‐210. doi:10.1016/s0360-3016(98)00544-6 10219815

[11] Kamada T , Tsujii H , Blakely EA , et al. Carbon ion radiotherapy in Japan: an assessment of 20 years of clinical experience. Lancet Oncol. 2015;16(2):e93‐e100. doi:10.1016/S1470-2045(14)70412-7 25638685

[12] Yokoi E , Mabuchi S , Takahashi R , et al. Impact of histological subtype on survival in patients with locally advanced cervical cancer that were treated with definitive radiotherapy: adenocarcinoma/adenosquamous carcinoma versus squamous cell carcinoma. J Gynecol Oncol. 2017;28(2):e19. doi:10.3802/jgo.2017.28.e19 28028992 PMC5323286

[13] Miyasaka Y , Yoshimoto Y , Murata K , et al. Treatment outcomes of patients with adenocarcinoma of the uterine cervix after definitive radiotherapy and the prognostic impact of tumor‐infiltrating CD8+ lymphocytes in pre‐treatment biopsy specimens: a multi‐institutional retrospective study. J Radiat Res. 2020;61(2):275‐284. doi:10.1093/jrr/rrz106 32052042 PMC7246070

[14] Minkoff D , Gill BS , Kang J , Beriwal S . Cervical cancer outcome prediction to high‐dose rate brachytherapy using quantitative magnetic resonance imaging analysis of tumor response to external beam radiotherapy. Radiother Oncol. 2015;115(1):78‐83. doi:10.1016/j.radonc.2015.03.007 25805517

[15] Wakatsuki M , Kato S , Ohno T , et al. Clinical outcomes of carbon ion radiotherapy for locally advanced adenocarcinoma of the uterine cervix in phase 1/2 clinical trial (Protocol 9704). Cancer. 2014;120(11):1663‐1669. doi:10.1002/cncr.28621 24591084

[16] Okonogi N , Wakatsuki M , Kato S , et al. Clinical outcomes of carbon ion radiotherapy with concurrent chemotherapy for locally advanced uterine cervical adenocarcinoma in a phase 1/2 clinical trial (Protocol 1001). Cancer Med. 2018;7(2):351‐359. doi:10.1002/cam4.1305 29341491 PMC5806111

[17] Okonogi N , Ando K , Murata K , et al. Multi‐institutional retrospective analysis of carbon‐ion radiotherapy for patients with locally advanced adenocarcinoma of the uterine cervix. Cancer. 2021;13(11):2713. doi:10.3390/cancers13112713 PMC8198465 34072676

[18] Okonogi N , Fukahori M , Wakatsuki M , et al. Dose constraints in the rectum and bladder following carbon‐ion radiotherapy for uterus carcinoma: a retrospective pooled analysis. Radiat Oncol. 2018;13:119. doi:10.1186/s13014-018-1061-7 29941040 PMC6019512

[19] Okonogi N , Saitoh J , Suzuki Y , et al. Changes in bone mineral density in uterine cervical cancer patients after radiation therapy. Int J Radiat Oncol Biol Phys. 2013;87(5):968‐974. doi:10.1016/j.ijrobp.2013.08.036 24139516

[20] Miyasaka Y , Okonogi N , Fukahori M , et al. Pelvic insufficiency fractures following carbon‐ion radiotherapy for uterine carcinomas. Radiother Oncol. 2021;156:56‐61. doi:10.1016/j.radonc.2020.11.030 33278405

[21] Ohno T , Kato S , Sato S , et al. Long‐term survival and risk of second cancers after radiotherapy for cervical cancer. Int J Radiat Oncol Biol Phys. 2007;69(3):740‐745. doi:10.1016/j.ijrobp.2007.04.028 17889265

[22] Mohamad O , Tabuchi T , Nitta Y , et al. Risk of subsequent primary cancers after carbon ion radiotherapy, photon radiotherapy, or surgery for localised prostate cancer: a propensity score‐weighted, retrospective, cohort study. Lancet Oncol. 2019;20(5):674‐685. doi:10.1016/S1470-2045(18)30931-8 30885458

[23] Okonogi N , Kobayashi D , Suga T , et al. Human papillomavirus genotype affects metastatic rate following radiotherapy in patients with uterine cervical cancer. Oncol Lett. 2018;15(1):459‐466. doi:10.3892/ol.2017.7327 29387229 PMC5769372

[24] Charlson ME , Pompei P , Ales KL , MacKenzie CR . A new method of classifying prognostic comorbidity in longitudinal studies: development and validation. J Chronic Dis. 1987;40(5):373‐383. doi:10.1016/0021-9681(87)90171-8 3558716

[25] National Cancer Registry (Ministry of Health, Labor and Welfare), tabulated by Cancer Information Service, National Cancer Center, Japan . Cancer Statistics in Japan 2. Incidence. https://ganjoho.jp/reg_stat/statistics/data/dl/en.html

[26] Kanda Y . Investigation of the freely available easy‐to‐use software ‘EZR’ for medical statistics. Bone Marrow Transplant. 2013;48(3):452‐458. doi:10.1038/bmt.2012.244 23208313 PMC3590441

[27] Berrington de Gonzalez A , Curtis RE , Kry SF , et al. Proportion of second cancers attributable to radiotherapy treatment in adults: a cohort study in the US SEER cancer registries. Lancet Oncol. 2011;12(4):353‐360. doi:10.1016/S1470-2045(11)70061-4 21454129 PMC3086738

[28] Arai T , Nakano T , Fukuhisa K , et al. Second cancer after radiation therapy for cancer of the uterine cervix. Cancer. 1991;67(2):398‐405. doi:10.1002/1097-0142(19910115)67:2<398::aid-cncr2820670214>3.0.co;2-a 1985734

[29] Wang L , Wang X , Zhang Q , et al. Is there a role for carbon therapy in the treatment of gynecological carcinomas? A systematic review. Future Oncol. 2019;15(26):3081‐3095. doi:10.2217/fon-2019-0187 31426679

[30] Li C , Zhang Q , Li Z , et al. Efficacy and safety of carbon‐ion radiotherapy for the malignant melanoma: a systematic review. Cancer Med. 2020;9(15):5293‐5305. doi:10.1002/cam4.3134 32524777 PMC7402834

[31] Arnold M , Liu L , Kenter GG , Creutzberg CL , Coebergh JW , Soerjomataram I . Second primary cancers in survivors of cervical cancer in The Netherlands: implications for prevention and surveillance. Radiother Oncol. 2014;111(3):374‐381. doi:10.1016/j.radonc.2014.04.011 24833558

[32] Damen JA , Hooft L , Schuit E , et al. Prediction models for cardiovascular disease risk in the general population: systematic review. BMJ. 2016;353:i2416. doi:10.1136/bmj.i2416 27184143 PMC4868251

[33] Zhao Q , Meng M , Kumar R , et al. The impact of COPD and smoking history on the severity of COVID‐19: a systemic review and meta‐analysis. J Med Virol. 2020;92(10):1915‐1921. doi:10.1002/jmv.25889 32293753 PMC7262275

[34] Schaapveld M , Aleman BM , van Eggermond AM , et al. Second cancer risk up to 40 years after treatment for Hodgkin's Lymphoma. N Engl J Med. 2015;373(26):2499‐2511. doi:10.1056/NEJMoa1505949 26699166

[35] Dertinger SD , Avlasevich SL , Torous DK , et al. Persistence of cisplatin‐induced mutagenicity in hematopoietic stem cells: implications for secondary cancer risk following chemotherapy. Toxicol Sci. 2014;140(2):307‐314. doi:10.1093/toxsci/kfu078 24798381 PMC4176048

[36] Lee B , Ahn SH , Kim H , et al. Secondary cancer‐incidence risk estimates for external radiotherapy and high‐dose‐rate brachytherapy in cervical cancer: phantom study. J Appl Clin Med Phys. 2016;17(5):124‐132. doi:10.1120/jacmp.v17i5.6087 PMC5874128 27685104

[37] Newhauser WD , Durante M . Assessing the risk of second malignancies after modern radiotherapy. Nat Rev Cancer. 2011;11(6):438‐448. doi:10.1038/nrc3069 21593785 PMC4101897

[38] Maraldo MV , Jørgensen M , Brodin NP , et al. The impact of involved node, involved field and mantle field radiotherapy on estimated radiation doses and risk of late effects for pediatric patients with Hodgkin lymphoma. Pediatr Blood Cancer. 2014;61(4):717‐722. doi:10.1002/pbc.24861 24660228

[39] Furukawa T , Inaniwa T , Sato S , et al. Performance of the NIRS fast scanning system for heavy‐ion radiotherapy. Med Phys. 2010;37(11):5672‐5682. doi:10.1118/1.3501313 21158279

[40] Matsumoto S , Yonai S . Evaluation of neutron ambient dose equivalent in carbon‐ion radiotherapy with energy scanning. Radiat Prot Dosimetry. 2020;191(3):310‐318. doi:10.1093/rpd/ncaa166 33111136

[41] Best T , Li D , Skol AD , et al. Variants at 6q21 implicate PRDM1 in the etiology of therapy‐induced second malignancies after Hodgkin's lymphoma. Nat Med. 2011;17(8):941‐943. doi:10.1038/nm.2407 21785431 PMC3229923

