# Supplementary material for: Secondary cancers after carbon‐ion radiotherapy and photon beam radiotherapy for uterine cervical cancer: A comparative study
Source: Cancer Med. 2022 Mar 23;11(12):2445–54. doi: 10.1002/cam4.4622 (PMC9189463; doi:10.1002/cam4.4622)
Supplement: Supplementary file 1 — Table S1‐S2 [file CAM4-11-2445-s002.docx]

**Supplementary table 1.** **List of secondary cancers patients after (A) photon radiotherapy and (B) carbon-ion radiotherapy.**

**(A) Photon radiotherapy**

| Case | Age at irradiation (years) | Second cancers | Relationship to irradiated sites | Latent period (years) | Calendar year of treatment | Prognosis |
| --- | --- | --- | --- | --- | --- | --- |
| 1 | 88 | stomach | outside | 0.4 | 2009 | died |
| 2 | 66 | stomach | outside | 0.5 | 2004 | alive |
| 3 | 80 | bile duct | outside | 0.7 | 2009 | alive |
| 4 | 82 | liver | outside | 0.7 | 2006 | died |
| 5 | 77 | pancreas | outside | 0.9 | 2006 | died |
| 6 | 84 | lung | outside | 1.0 | 2006 | alive |
| 7 | 74 | lung | outside | 1.1 | 2000 | died |
| 8 | 80 | thyroid | outside | 1.6 | 2012 | alive |
| 9 | 75 | liver | outside | 1.9 | 2002 | died |
| 10 | 74 | lung | outside | 2.0 | 2008 | alive |
| 11 | 80 | stomach | outside | 2.3 | 2004 | died |
| 12 | 64 | lung | outside | 2.4 | 2006 | alive |
| 13 | 59 | stomach | outside | 2.5 | 1998 | died |
| 14 | 76 | finger | outside | 2.6 | 1996 | died |
| 15 | 69 | lung | outside | 3.3 | 1995 | died |
| 16 | 87 | colon | inside | 3.7 | 2006 | died |
| 17 | 68 | breast | outside | 3.9 | 2003 | alive |
| 18 | 73 | cecum | inside | 4.1 | 2007 | alive |
| 19 | 59 | rectum | inside | 4.6 | 2003 | alive |
| 20 | 80 | stomach | outside | 5.5 | 2006 | alive |
| 21 | 87 | lung | outside | 5.7 | 1997 | died |
| 22 | 51 | bladder | inside | 6.6 | 2010 | alive |
| 23 | 61 | soft tissue (pelvis) | inside | 7.5 | 2005 | died |
| 24 | 69 | stomach | outside | 9.4 | 1996 | died |
| 25 | 78 | cecum | inside | 10.3 | 1999 | alive |
| 26 | 76 | hypopharynx | outside | 10.5 | 1998 | died |
| 27 | 49 | ovary | inside | 10.9 | 2006 | alive |
| 28 | 55 | bladder | inside | 20.6 | 1997 | alive |

**(B) Carbon-ion radiotherapy**

| Case | Age at irradiation (years) | Second cancers | Relationship to irradiated sites | Latent period (years) | Calendar year of treatment | Prognosis |
| --- | --- | --- | --- | --- | --- | --- |
| 1 | 76 | lung | outside | 2.5 | 2011 | alive |
| 2 | 36 | bladder | inside | 2.8 | 2013 | alive |
| 3 | 64 | plasmacytoma (pelvis) | inside | 3.3 | 2014 | died |
| 4 | 79 | colon | inside | 5.1 | 2011 | died |
| 5 | 76 | uterine | inside | 8.4 | 1997 | died |
| 6 | 43 | breast | outside | 9.0 | 2002 | alive |
| 7 | 57 | ovary | inside | 17.1 | 2000 | died |
| 8 | 61 | uterine (sarcoma) | inside | 17.3 | 2000 | died |
| 9 | 48 | soft tissue (pelvis) | inside | 22.4 | 1995 | alive |

**Supplementary table 2. Characteristics in each group of calendar year of treatment.**

|  | **Calendar year of treatment**  **(1995**–**2005)** | **Calendar year of treatment**  **(2006**–**2016)** | **P-value** |
| --- | --- | --- | --- |
| **Age** |  |  |  |
| **>62** | 181 | 122 | 0.358 |
| **≤62** | 198 | 113 |  |
| **Time to development of secondary cancer (year)**  **Median (IQR)** | 6.6 (2.6–10.4) | 2.5 (1.0–4.1) | 0.014 |

Abbreviation: IQR = Interquartile range.
