# Supplementary figures and images for: Secondary cancers after carbon‐ion radiotherapy and photon beam radiotherapy for uterine cervical cancer: A comparative study
Source: Cancer Med. 2022 Mar 23;11(12):2445–54. doi: 10.1002/cam4.4622 (PMC9189463; doi:10.1002/cam4.4622)

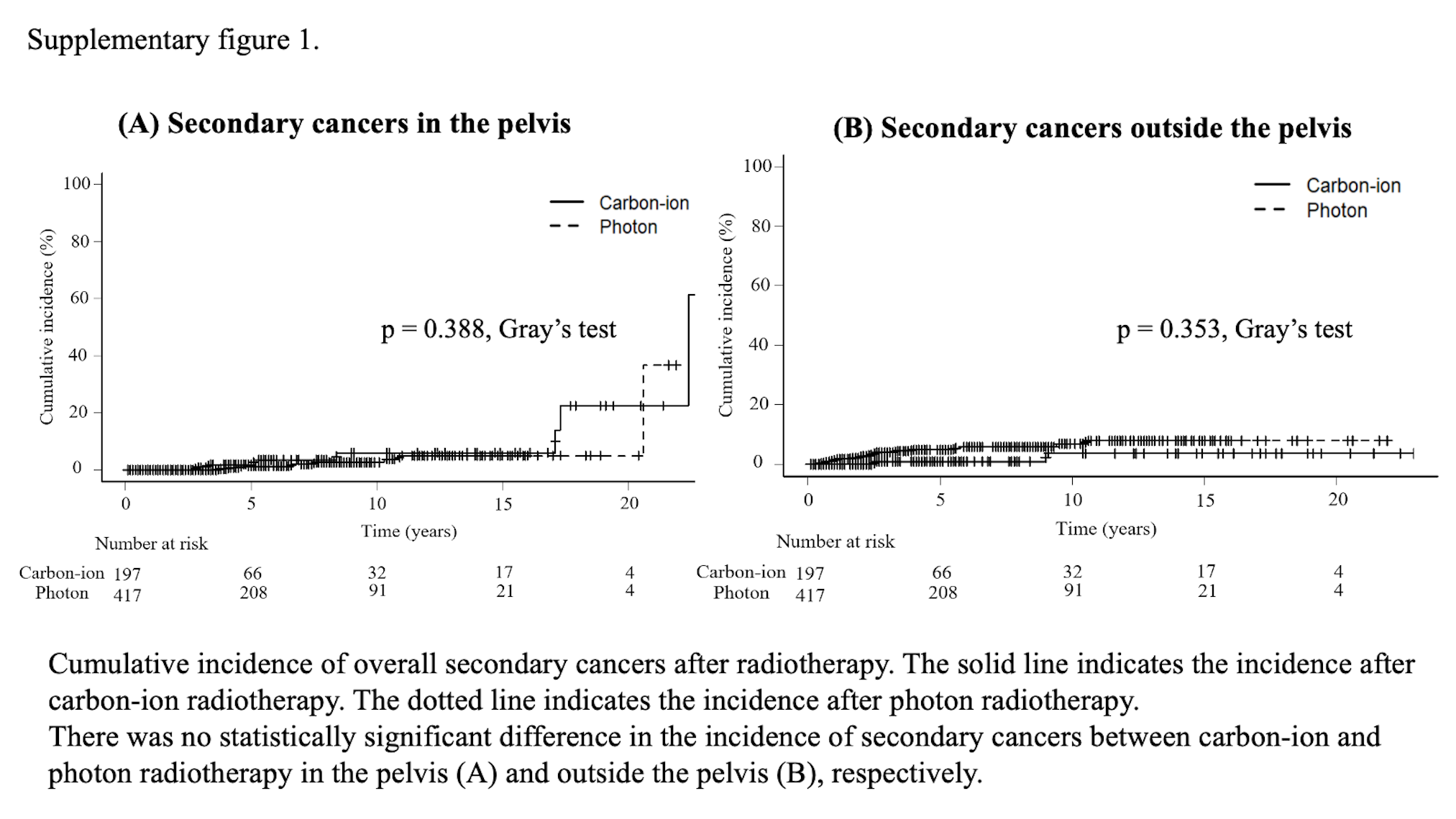

Supplement: Supplementary file 2 — Figure S1 [file CAM4-11-2445-s001.tiff]
